# Burnout scales used in healthcare workers during the COVID-19 pandemic and their psychometric properties: a systematic review

**DOI:** 10.3389/fpubh.2025.1661548

**Published:** 2026-01-08

**Authors:** Maria Igorevna Efremova, Yeajoon Cho, Elisabetta Miglietta, Mariana Pinto da Costa

**Affiliations:** 1 Institute of Psychiatry, Psychology & Neuroscience, King’s College London, London, United Kingdom; 2 South London and Maudsley NHS Foundation Trust, London, United Kingdom; 3 European University Institute, Fiesole, Italy

**Keywords:** burnout, COVID-19 pandemic, healthcare professionals, psychometrics (MeSH), scales (MeSH)

## Abstract

**Background:**

Burnout has been assessed by a variety of screening instruments worldwide. This systematic review investigated the characteristics and psychometric properties of these scales used during the COVID-19 pandemic to assess burnout among healthcare workers.

**Methods:**

A systematic literature search and review was conducted based on four operational criteria: burnout scales, healthcare workers, psychometric properties, and COVID-19. We retrieved records from APA PsycInfo, APA PsycArticles, MEDLINE, Academic Search Complete, and EMBASE. All peer reviewed articles that assessed burnout in healthcare workers during COVID-19 were included.

**Results:**

A total of 794 articles met the inclusion criteria, and 27 burnout scales were identified, two of which were developed during the pandemic. The scales varied in number of items, subscales, response options, language, and country of development. At least one psychometric property was reported for 19 of the 27 scales, and 15 scales demonstrated desirable internal consistency in this novel context. For validity, 9 out of 27 scales reported at least one psychometric property. This was predominantly for measures on structural validity.

**Conclusion:**

Although a wide range of scales were used to assess burnout in healthcare workers during COVID-19, the psychometric properties reported were predominantly on reliability rather than validity. The findings of this review can be used to guide appropriate instrument selection for burnout in crisis contexts such as the COVID-19 pandemic.

**Systematic review registration:**

PROSPERO registration database: CRD42023414070.

## 1 Introduction

The outbreak of the COVID-19 pandemic has affected communities and occupations worldwide, particularly frontline key workers. Among these, healthcare workers, including, but not limited to, doctors, nurses, physicians and pharmacists, have experienced a substantial impact (1). Healthcare workers were faced with an increased fear of infection (2), moral dilemmas associated with delivering optimal care to an overwhelming number of patients (3), shortages of personal protective equipment (4), and staff shortages due to colleagues needing to quarantine (5). Consequently, the novel demands, challenges and resource shortages have generated scholarly interest in investigating COVID-related effects within the healthcare professions. This encompassed investigating the impact on outcomes such as post-traumatic stress (6), insomnia (7), depression (8), and physical health (9). As the burden on healthcare workers increased during the COVID-19 pandemic, a high number of studies were published on this occupational group, reporting high levels of burnout (10).

Burnout has been characterised by three dimensions: feelings of energy depletion or exhaustion; increased mental distance from one’s job, or feelings of negativism or cynicism related to one’s job; and a sense of ineffectiveness and lack of accomplishment (11). Burnout refers specifically to phenomena in the occupational context and should not be applied to describe experiences in other areas of life (12). Reports concerning healthcare workers during the COVID-19 pandemic have revealed that an alarmingly high number of individuals were experiencing burnout with rates as high as 43% (13). Prior to the COVID-19 pandemic, burnout rates in healthcare workers varied but rates as low as 2.5% were reported in a systematic review (14). This phenomenon has been associated with increased rates of sickness absences (15), underperformance (16), and quitting their jobs (17). Previous studies in this area have underscored the policy implications, highlighting the necessity of implementing coping strategies in similar critical situations in the future (18). It has been emphasised that future research should focus on elucidating the factors influencing mental health, especially during critical times such as the COVID-19 pandemic (8).

Despite the increased widespread attention to burnout within the healthcare profession during the COVID-19 pandemic, understanding of the general characteristics and psychometric properties of the burnout scales used in this area of research remains limited. For example, a systematic review of burnout prevalence during the COVID-19 pandemic showed that the results could differ depending on whether a full or a modified scale was used (19). With regards to psychometrics for burnout scales, there has been only one systematic review to date, conducted before the COVID-19 pandemic, which recognised insufficiencies in the psychometric properties of assessed burnout scales and called for the development of a diagnostic standard for burnout (20).

The COVID-19 pandemic brought an array of unpredictable and unforeseen challenges, such as medical supply shortages and limited access to personal protective equipment that have been associated with increased rates of burnout in healthcare workers (21). Given the unique pandemic-specific emergency context and the pressures faced by healthcare workers, these novel factors may not have been adequately captured by the previously used measures of burnout. In response to this, new scales specific to the COVID-19 context were developed (22, 23). While established instruments for measuring burnout continue to be used in occupational contexts that affect the wellbeing of healthcare workers, including during crises (19, 24), it is crucial to assess their characteristics and psychometric properties, with particular attention to domains of validity and reliability that are especially important for crisis contexts (20, 25). This work is essential because tools with evidence of psychometric rigour support accurate assessment, provide confidence in the detection of rapid or severe fluctuations, and enable timely targeted interventions and policy decisions which are grounded in a robust evidence base (26).

Therefore, this study aimed: (i) to investigate what measures have been used to assess burnout in healthcare workers during the COVID-19 pandemic; (ii) to report on structure and characteristics of these measures, and their psychometric properties; and (iii) to inform which scales are more recommendable to assess burnout in healthcare workers in relation to the COVID-19 pandemic.

## 2 Methodology

### 2.1 Search strategy

This systematic review adopted the Preferred Reporting Items for Systematic Reviews and Meta-Analyses (PRISMA) (27). The study was registered in the PROSPERO database (CRD42023414070). The following online databases were searched: APA PsycInfo (through OVID), APA PsycArticles (through OVID), MEDLINE (through OVID), Academic Search Complete (through OVID), and EMBASE (through EBSCOhost); up to March 19th, 2023. There were no restrictions with regards to language. The search terms were refined through a series of searches in the aforementioned databases to test the use of truncation “*.” The final search terms were as follows: measure* OR instrument* OR questionnaire* OR test* OR measurement* OR scale* OR survey* OR self-report* OR validity OR reliability OR psychometric* OR internal consistency OR test–retest reliability OR construct validity OR convergent validity OR discriminant validity OR criterion validity AND (depersonal* OR personal accomplishment OR physical exhaustion OR mental exhaustion OR exhaustion OR disengagement OR exhaustion OR professional exhaustion OR burnout OR burn out) AND (doctor* OR general practitioner OR GP OR therapist* OR nurs* OR paramedic* OR health professional* OR medical staff OR health* practitioner* OR health*assistants OR health* workers OR medical consultant* OR dentist* OR midwi* OR art therapist* OR drama therapist* OR music therapist* OR podiatrist* OR dietitian* OR occupational therapist* OR operating department practitioner* OR orthoptist* OR osteopath* OR physiotherapist* OR prosthetist* OR orthotist* OR radiographer* OR speech and language therapist* OR clinical psychologist OR psychologist OR counsel* OR paediatric* OR surgeon OR anaesthetist OR cardiologist OR gynaecologist OR emergency medicine specialist OR ophthalmologist OR obstetrician pathologist OR psychiatrist OR physician OR radiologist OR dental* OR audiologist OR pharmaceutical OR ambulance worker OR first-aid attendant) AND (covid-19 OR pandemic OR coronavirus OR SARS-CoV OR covid*).

### 2.2 Operational definitions

#### 2.2.1 Presence of a burnout scale

The inclusion of a burnout scale in this systematic review was determined by the instruments in the examined empirical research. These instruments assessed emotional and mental exhaustion, cynicism, depersonalization, professional inefficacy, reduced personal accomplishment, and disengagement. This criterion is consistent with the literature on the burnout construct and its various definitions (28).

#### 2.2.2 Healthcare workers

The criteria used to identify healthcare workers in the articles analysed for this systematic review were adopted from the World Health Organisation (29). In line with the WHO classification, we included all healthcare workers in the occupational groups ‘Health Professionals’ and ‘Health Associate Professionals’, including students training to become healthcare professionals, and excluding healthcare workers involved in Traditional and Complementary Medicine.

#### 2.2.3 COVID-19 pandemic

We conducted our search without any specific date restrictions, which enabled us to retrieve all relevant literature related to the COVID-19 pandemic published up to the date of search on 19^th^ March 2023. We captured all potential variations of COVID-19, including terms like pandemic, coronavirus and SARS-CoV.

### 2.3 Eligibility criteria

We included studies that met the following criteria: (i) related to burnout among healthcare workers during the COVID-19 pandemic; (ii) included at least one type of self-report scale or subscale for assessing burnout; (iii) used quantitative research methods, which included an assessment of burnout; (iv) were published in peer-reviewed journals. Records were excluded if they included a burnout scale but lacked psychometric properties or references to a validation article. Furthermore, records were excluded if they included healthcare workers albeit merged with other samples. However, if a study included healthcare workers and other samples that were analysed separately then they were included. There were no exclusion criteria regarding the location or type of practice (private or public) of healthcare workers.

### 2.4 Data extraction and screening procedures

One author searched and exported all retrieved records to EndNote 20 (Clarivate Analytics, EndNote 20, 2021). Initially, duplicate records were identified and removed using the EndNote software. Subsequently, any remaining duplicates, not identified by EndNote, were manually screened and removed by two authors in Excel. Two of the authors independently evaluated the articles by title and abstracts according to the inclusion criteria. Any discrepancies were resolved by the other two authors. The full texts were then screened by two authors for eligibility according to the inclusion criteria. Any discrepancies were resolved by the two other authors.

### 2.5 Scales selection and psychometric properties extraction

Records that included at least one burnout scale in their research were included. If the empirical studies conducted during the COVID-19 pandemic included reports on the psychometric properties of the burnout scales (such as reliability and validity), we extracted these details. If there were no psychometric properties reported, we referred to the original development and validation articles to obtain the scales’ psychometric properties. The guidelines for validating the psychometric properties outlined by were followed.

## 3 Results

### 3.1 Literature search

**Figure 1:**
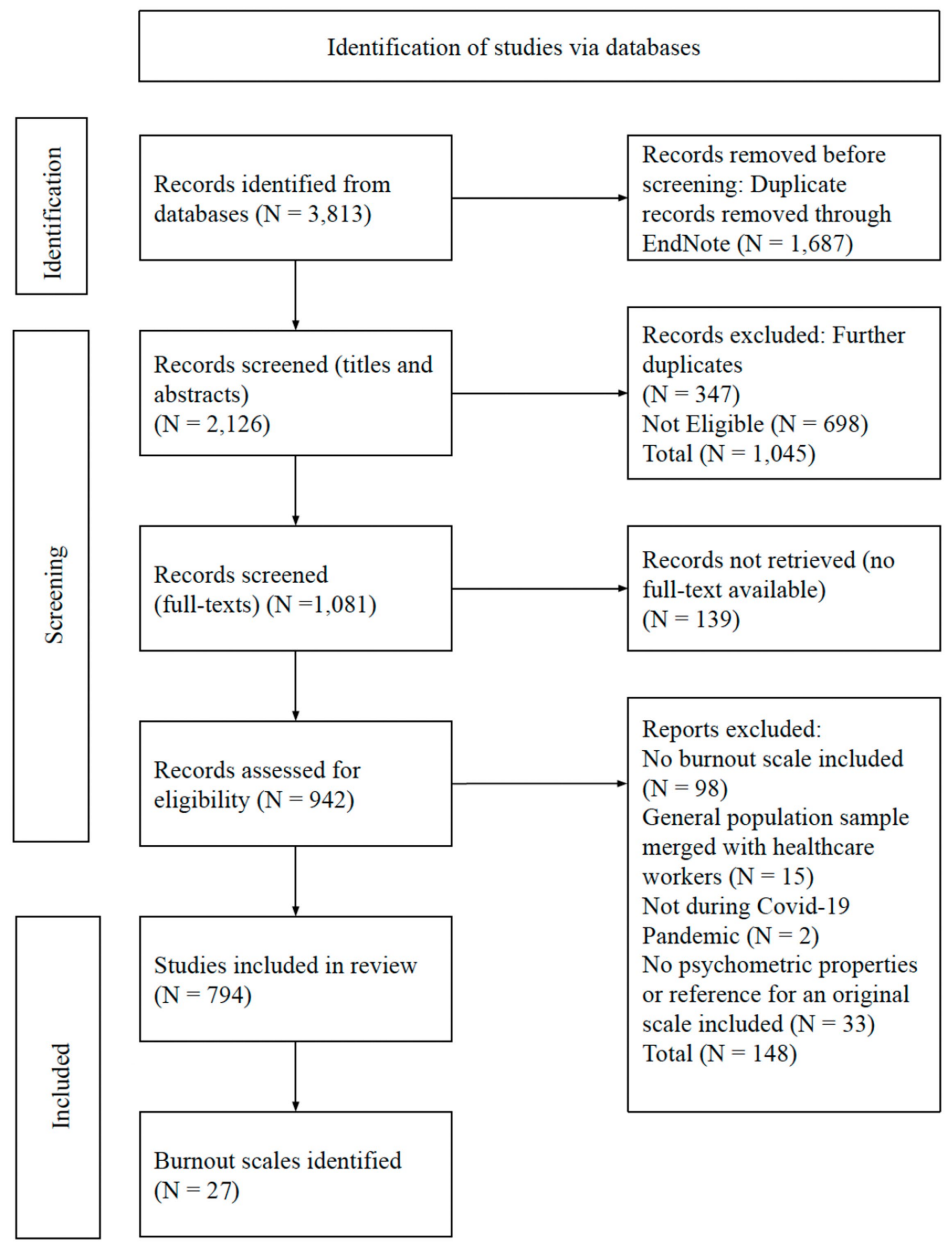
PRISMA flow chart of selecting included studies and scales.

A total of 3,813 records were retrieved in the electronic search. After removing duplicates (*N =* 1,687) using EndNote 20 (Clarivate Analytics, EndNote 20, 2021), the total number of records screened for title and abstract eligibility was established (*N =* 2,126). During this screening phase, 1,045 records were excluded for two reasons: additional duplicates (*N =* 347) and records that did not meet our eligibility criteria (*N =* 698). The remaining set comprised 1,081 research studies, which were screened for full-text availability. The total number of records that were not available was 139. Therefore, a total of 942 full-text records were screened and assessed according to the outlined above eligibility criteria. After full-text screening, the total number of articles excluded was 148. Thus, 794 full-text records included at least one burnout scale and were therefore included in this review. All of these 794 full-text records were based on research undertaken during the COVID-19 pandemic on healthcare workers and spanned across 54 countries. Figure 1 presents the PRISMA flow chart for this study, including a more detailed summary of the study selection process and details the reasons for excluding the records at each stage of the eligibility screening and assessment.

### 3.2 General characteristics

We identified 27 scales that were used to assess burnout among healthcare workers during the COVID-19 pandemic. All scales identified were self-reported measures. Eighteen of these scales represented scales primarily used to assess burnout, while the rest of the scales were multi-dimensional measures which included a burnout component as part of a secondary subscale (*N* = 9).

The number of items of the included scales ranged from 1 to 127, and all the scales adopted a Likert-scale response format, with the majority using a scale ranging from 1 (never) to 5 (everyday). One scale also used a dichotomous rating of ‘Yes/No’ (30). Out of the 18 scales which comprised subscales, the number of subscales ranged from two to 41. The number of items specifically measuring burnout for the primary burnout scales was 2 to 26; for the multi-dimensional scales including a burnout component secondarily, this was 1 to 17. The number of subscales for the primary burnout scales ranged from one to four.

The majority of scales were originally developed in the United States of America (*N =* 15); the others were developed in Denmark (*N =* 2), Israel (*N =* 2), Spain (*N =* 2), Belgium (*N =* 1), Finland (*N* = 1), Germany (*N* = 1), Greece (*N* = 1), Japan (*N* = 1), and Turkey (*N* = 1).

Different translations were used for the 19 of the scales measuring burnout. Maslach Burnout Inventory-Human Services Survey (MBI-HSS) had the highest number of validated translations with 30 different language versions identified on this review. The most popular language used to assess burnout was in English, with 258 of the identified studies (out of 794 studies) using the English versions of the scale.

### 3.3 Characteristics of scales used primarily to assess burnout

Out of the 18 scales used to primarily assess burnout, five of these were different versions of MBI-HSS. Though related, Maslach Burnout Inventory-General Survey (MBI-GS; 16-item) was treated as a distinct version from MBI-HSS (22-item) as it differed in its primary target population as well as the number of items and dimensions of burnout measured.

For MBI-HSS, the versions differed according to the number of items but also the subscales included, reflecting different dimensions of burnout measured. For instance, the MBI-EE (9-item) version represents the full subscale of the original MBI-HSS measuring the ‘emotional exhaustion’ dimension, whereas aMBI is an ‘abbreviated’ measure of MBI-HSS that encompasses all three dimensions of the original scale – three items each from each dimension of ‘emotional exhaustion’ (MBI-EE), depersonalisation (MBI-DP), and ‘professional accomplishment’ (MBI-PA).

Two versions of MBI-HSS (MBI-EE: 2-item, and MBI-EE/DP: 2-item) represent significantly shortened subscales of the MBI-HSS comprising of one/two-item(s) measures for ‘emotional exhaustion’ and one-item measure for ‘depersonalisation’ which should be best treated as screening tools for the dimensions of burnout probed.

All 18 scales included the ‘exhaustion’ dimension of burnout with ‘emotional exhaustion’ being the most specified form of exhaustion measured (*N* = 10). A total of 13 scales contained more than one dimension of burnout, and the number of dimensions measured varied from one to four. Out of the five scales consisting of only one burnout dimension, three scales measured ‘emotional exhaustion’ (MBI-EE: 9-item, MBI-EE: 5-item, MBI-EE: 2-item) whereas two scales provided a unidimensional measure of exhaustion encompassing physical, emotional and mental aspects [Burnout Measure-Short version (BMS), and COVID-19 Burnout Scale: 10-item].

There were two scales which were developed during the COVID-19 pandemic (22, 23). They are both referred to as the COVID-19 Burnout Scale and are differentiated initially by the number of items and the original author references. The 10-item version by Yildrim & Solmaz represents a COVID-19 specific adaptation of the BMS (Malach-Pines, 2005) where a COVID-19 specifier has been added to all items of the BMS for contextualisation. In contrast, the 13-item version by Galanis et al. comprises of a unique (four-item) dimension measuring the ‘exhaustion due to measures against COVID-19.’

### 3.4 Characteristics of multi-dimensional scales including a secondary subscale of burnout

Out of the nine scales in this group, three scales (Compassion Fatigue Self-Test, Compassion Fatigue Short Scale, Professional Quality of Life-Version 5) measured burnout in the context of compassion fatigue which is defined as a combined measure of burnout and secondary traumatic stress.

Copenhagen Psychosocial Questionnaire-Version II (COPSOQ-II) used the ‘personal burnout’ scale adapted from CBI as part of an assessment for psychosocial factors in the work environment, while the PFI scale measured burnout dimensions in ‘work exhaustion’ and ‘disengagement’ to assess for professional well-being.

The rest of the four scales (Well-Being Index, Physician Works Life Study, Mini-Z Survey, Mini-Z one-item) all used a single-item measure of burnout which have a very similarly worded question and responses that are ordered on a 5-point Likert (ordinal) scale. Practically, they could be treated as the same screening measure for burnout reflecting minor differences in their development and validation articles.

The Physician Work Life Study (PWLS) and Mini-Z one-item represent functionally similar versions of a single-item burnout measure which are incorporated into WBI to give a measure of well-being and into Mini-Z Survey to provide assessment of burnout prevalence and further screening for work-related stressors in healthcare workers. This one-item burnout measure has demonstrated good correlation with the ‘emotional exhaustion’ dimension of burnout.

### 3.5 Healthcare workers

All burnout scales, except for one Well-Being Index (WBI), were originally developed to assess burnout within the general population and did not specifically target healthcare workers. The WBI was specifically developed to assess both wellbeing and burnout among medical students, pharmacists, physicians, nurses and dentists (30).

The healthcare workers in the identified articles were referred to by an array of diverse professional titles. Table 1 shows the variety of terms used to refer to the target healthcare workers. While this list is not exhaustive of all terms identified, it outlines the range encountered across the studies. Novel terms relating to ‘COVID-19’, such as ‘COVID-19 dedicated nurses’, reflect the pandemic context. ‘Healthcare workers’ was the term most encountered.

**Table 1:** Summary of burnout scales identified during the systematic review.

Scale name	Other versions	Original reference	Country of development	Developed during COVID-19	Number of COVID-19 articles	Versions of translated scales (N) identified in the review	Target Healthcare workers
Scales used primarily to assess burnout							
MBI-HSS (22-item)		Maslach & Jackson (1981)	USA	No	333	English (*N =* 74), Italian (*N =* 43), Turkish (*N =* 38), Spanish (*N =* 34), Persian (*N =* 21), Portuguese (*N =* 18), Chinese (*N =* 16), Arabic (*N =* 14), French (*N =* 12), Polish (*N =* 8), Japanese (*N =* 6), Greek (*N =* 5), Urdu (*N =* 5), German (*N =* 4), Korean (*N =* 4), Romanian (*N =* 4), Dutch (*N =* 3), Indonesian (3), Hebrew (*N =* 2), Hungarian (*N =* 2), Malay (*N =* 2), Serbian (*N =* 2), Bengali (*N =* 1), Czech (*N =* 1), Kazakh (*N =* 1), Russian (*N =* 1), Slovak (*N =* 1), Syrian (*N =* 1), Thai (*N =* 1), Twi (*N =* 1), Multiple (*N =* 5)	Healthcare workers, Frontline healthcare workers, Nursing home workers, Long-term care workers, Mental health workers, Healthcare professionals, Primary care professionals, Palliative care professionals, Nursing professionals, Addiction treatment providers, Nephrology workforce Physicians (Specialists physicians, ICU specialists, Oncologists, Paediatricians, GPs, Residents, Interns etc.) Nurses, Frontline nurses, Emergency nurses, ICU nurses, Midwives, Nursing assistants etc.), EMTs, Radiographers, Radiation therapy technologists, Pharmacists, Community pharmacists, Psychologists
	MBI-EE (9-item)	Maslach & Jackson (1981)	USA	No	16	English (*N =* 3), Italian (*N =* 3), French (*N =* 2), German (*N =* 2), Chinese (*N =* 1), Hungarian (*N =* 1), Korean (*N =* 1), Romanian (*N =* 1), Spanish (*N =* 1), Taiwanese (*N =* 1)	Healthcare workers, Healthcare professionals, Emergency workers, Workers in COVID-19 vaccination centres, Nurses, Employees from paediatric developmental services
	MBI-EE (5-item)	Maslach & Jackson (1981)	USA	No	4	Italian (*N =* 2), Romanian (*N =* 1), Chinese (*N =* 1)	Healthcare workers
	MBI-EE (2-item)	Maslach & Jackson (1981)	USA	No	8	English (*N =* 6), Italian (*N =* 1), Multiple (*N =* 1)	Healthcare workers, Family physicians, Nurses, Psychotherapists
	MBI-EE, MBI-DP (2-item)	West et al. (2009)	USA	No	15	English (*N =* 10), French (*N =* 1), Portuguese (*N =* 1), Russian (*N =* 1), Multiple (*N =* 2)	Healthcare workers, Frontline healthcare workers, Medical trainees, Nurses
	aMBI	Maslach et al. (1997)	USA	No	21	English (*N =* 16), Arabic (*N =* 2), Japanese (*N =* 1), Portuguese (*N =* 1), Multiple (*N =* 1)	Healthcare workers, Frontline healthcare workers, Physicians, Family physicians, Senior doctors, Neurosurgeons, Surgical trainees, Psychiatry trainees, Paramedics
MBI-GS (16-item)		Maslach et al. (1996)	USA	No	60	Chinese (*N =* 28), Italian (*N =* 5), English (*N =* 4), Japanese (*N =* 4), Korean (*N =* 4), Romanian (*N =* 4), Spanish (*N =* 3), Russian (*N =* 2), Arabic (*N =* 1), Polish (*N =* 1), Portuguese (*N =* 1), Taiwanese (*N =* 1), Thai (*N =* 1), Ukrainian (*N =* 1)	Healthcare workers, Frontline medical staff, Intensive care physicians, Doctors, Residents, Nurses, Frontline nurses, ICU nurses, Midwives
OLBI (16-item)		Demerouti & Nachreiner (1998)	Germany	No	40	English (*N =* 14), Polish (*N =* 6), Portuguese (*N =* 6), French (*N =* 4), Croatian (*N =* 2), Persian (*N =* 2), Greek (*N =* 1), Korean (*N =* 1), Malay (*N =* 1), Swedish (*N =* 1), Taiwanese (*N =* 1), Multiple (*N =* 1)	Healthcare workers, Geriatric healthcare workers ICU staff, Emergency department staff, Radiation oncology professionals, Medical employees in psychiatric care Physicians, Emergency physicians, Doctors, Anaesthesiologists, Oncologists, Oncology fellows, Gynaecologists, Psychiatrists, GPs, Residents, Interns, Trainees, Nurses, School nurses, Pharmacists, Psychologists, Obstetric sonographers
CBI (19-item)		Kristensen et al. (2005)	Denmark	No	81	English (*N =* 23), Arabic (*N =* 12), Portuguese (*N =* 11), Taiwanese (*N =* 4), Turkish (*N =* 4), Chinese (*N =* 3), Greek (*N =* 3), Italian (*N =* 3), Serbian (*N =* 3), Malay (*N =* 2), Persian (*N =* 2), Romanian (*N =* 2), Bosnian (*N =* 1), Danish (*N* = 1), French (*N =* 1), German (*N =* 1), Korean (*N =* 1), Thai (*N =* 1), Telugu (*N* = 1), Multiple (*N =* 2)	Healthcare workers, Frontline healthcare workers, Primary healthcare workers, Mental healthcare workers, Community health workers, Medical workers, Mental health services providers, Direct care providers, Healthcare professionals, Hospital staff, NHS staff, Emergency department staff, Physicians, Outpatient physicians, Primary care physicians, Family physicians, GPs, Specialists, Trainee anaesthetists, Doctors, Doctors in training, Postgraduate medical trainees, Gynaecologic surgery fellows, Paediatric urologists, Psychiatrists, Nursing and allied health staff, Nurses, midwives and allied health professionals, Nurses, Frontline nurses, Emergency nurses, Paediatric nurses, Nurse leaders, Nurse managers, Hospital nurses, EMTs, Paramedics, Pharmacy professionals, Pharmacy practitioners, Pharmacists, Community pharmacists, Physiotherapists, Neuropsychologists
BMS (10-item)	BMS (10-item)	Malach-Pines (2005)	Israel	No	16	English (*N =* 4), Turkish *N =* 4), Arabic (*N =* 2), Bengali (*N =* 2), Spanish (*N =* 2), Korean (*N =* 1), Urdu (*N =* 1)	Healthcare workers, Physicians, Resident doctors, Family medicine residents, Nurses, Student nurses, Dental hygienists, Community Pharmacists, Physiotherapists
SMBM (14-item)		Shirom & Melamed (2006)	Israel	No	8	English (*N =* 4), Hebrew (*N =* 2), Swedish (*N =* 1), Portuguese (*N =* 1)	Healthcare workers, Healthcare professionals, Hospital staff, Hospital care team, Otolaryngology residents, Nurses
CESQT (20-item)		Gil-Monte (2009)	Spain	No	1	Spanish (*N =* 1)	Nurses
BBI (9-item)		Salmela-Aro et al. (2010)	Norway	No	1	English (*N =* 1)	Frontline workers
Japanese Burnout Scale (17-item)		Kubo (1994)	Japan	No	2	Japanese (*N =* 2)	Physicians, Nurses
Granada Burnout Questionnaire (26-item)		Fuente et al. (2015)	Spain	No	1	Spanish (*N =* 1)	Nurses
BAT-C (23-item)		Schaufeli et al. (2020)	Belgium	No	9	German (*N =* 3), Dutch (*N =* 2), English (*N =* 1), Norwegian (*N =* 1), Polish (*N =* 1), Swedish (*N =* 1)	Healthcare workers, Healthcare professionals, Workers at a mental health institution, Clinicians, Physicians, Doctors, Nurses
COVID-19 Burnout Scale (10-item)		Yildirim & Solmaz (2022)	Turkey	Yes	3	English (*N =* 1), Thai (*N =* 1), Turkish (*N =* 1)	Health and social care workers, Healthcare staff, Healthcare personnel
COVID-19 Burnout Scale (13-item)		Galanis et al. (2022)	Greece	Yes	2	Greek (*N =* 2)	Nurses
Multi-dimensional scales including a secondary subscale of burnout							
CFST (40-item)		Figley (1995)	USA	No	1	English (*N =* 1)	Physicians
COPSOQ-II (full version comprises of 41 subscales totalling 127 items)		Kristensen et al. (2005)	Denmark	No	2	Persian (*N =* 1), Portuguese (*N =* 1)	Health care workers, Nurses
Compassion Fatigue Short Scale (13-item)		Adams et al. (2006)	USA	No	1	Turkish (*N =* 1)	Midwives
ProQOL-5 (30-item)		Stamm (2010)	USA	No	76	English (*N =* 27), Italian (*N =* 13), Spanish (*N =* 7), Arabic (*N =* 6), Chinese (*N =* 5), Greek (*N =* 5), Turkish (*N =* 3), French (*N =* 2), Georgian (*N =* 1), Hungarian (*N =* 1), Korean (*N =* 1), Persian (*N =* 1), Polish (*N =* 1), Portuguese (*N =* 1), Taiwanese (*N =* 1), Multiple (*N =* 1)	Healthcare workers, Healthcare professionals, Mental health workers, ICU healthcare professionals, Infection prevention professionals, Healthcare providers, Nursing care providers, Mental health practitioners, Infant and early child mental health workforce, Nurses working in COVID-19 area, COVID-19 dedicated nurses, Doctors, Internists, Radiologists, Dentists, Nurses, Frontline nurses, CCU nurses, HIV nurses, Nurse practitioners, Counsellors, Respiratory therapists, Radiologist, Imaging technologists
WBI (7-item)		Dyrbye et al. (2010)	USA	No	4	English (*N =* 4)	Healthcare workers, Healthcare professionals, Hospital workers, Clinicians
PWLS (1-item)		Dolan et al. (2015)	USA	No	39	English (*N =* 26), Portuguese (*N =* 3), Italian (*N =* 2), Mandarin (*N =* 2), Arabic (*N =* 1), French (*N =* 1), Norwegian (*N =* 1), Multiple (*N =* 3)	Healthcare workers, Healthcare professionals, Healthcare providers, Advanced practice providers, Healthcare practitioners, Emergency department personnel, Medical staff, Clinicians, Physicians, Frontline physicians, Residents, Gastroenterology trainees, Nurses, Frontline acute care nurses
Mini-Z Survey (10-item)		Linzer et al. (2016)	USA	No	13	English (*N =* 10), Japanese (*N =* 1), Portuguese (*N =* 1), Multiple (*N =* 1)	Healthcare workers, Head and neck surgeons, Physiotherapists, psychologists
	Mini-Z (1-item)	Linzer et al. (2016)	USA	No	14	English (*N =* 10), Japanese (*N =* 3), Spanish (*N =* 1)	Healthcare workers, Frontline healthcare workers, Hospital staff, Radiation oncology employees, Physicians, Rheumatologists, Otolaryngology residents
PFI (16-item)		Trockel et al. (2018)	USA	No	23	English (*N =* 19), French (*N =* 1), Italy (*N =* 1), Multiple (*N =* 2)	Healthcare workers, Healthcare providers, Hospice professionals, Mental health practitioners, Physicians, Frontline physicians, Anaesthesiologists, Doctors, Surgeons, Residents, Surgical trainees, Nurses

MBI-HSS, Maslach Burnout Inventory-Human Services Survey; MBI-EE, Maslach Burnout Inventory-Emotional Exhaustion; MBI-DP, Maslach Burnout Inventory-Depersonalisation; MBI-PA, Maslach Burnout Inventory-Professional Accomplishment; aMBI, Abbreviated Maslach Burnout Inventory; MBI-GS, Maslach Burnout Inventory-General Survey; OLBI, Oldenburg Burnout Inventory; CBI, Copenhagen Burnout Inventory; BMS, Burnout Measure-Short Version; SMBM, Shirom-Melamed Burnout Measure; CESQT, Spanish Burnout Inventory; BBI, Bergen Burnout Inventory; BAT-C, Burnout Assessment Tool-Core symptoms; CFST, Compassion Fatigue Self-Test; COPSOQ-II, Copenhagen Psychological Questionnaire-Version II; ProQOL-5, Professional Quality of Life-Version 5; WBI, Well-Being Index; PWLS, Physician Work Life Study; PFI, Professional Fulfillment index.

### 3.6 Psychometric properties

The summary of the psychometric properties of the burnout screening instruments identified in this systematic review are presented in Table 2. For 19 scales at least one form of psychometric property was reported in the COVID-19 pandemic context (22–24, 30–43). For the remaining eight, psychometric properties were not reported in the retrieved articles.

**Table 2:** Psychometric properties of validation and retrieved papers.

Scale name	Subscales	Number of items	Response option	Number of studies (N) reporting on psychometrics during COVID-19	Psychometric properties from studies during COVID-19	Psychometric properties from original validation papers
Scales used primarily to assess burnout						
MBI-HSS (22-item)	EE (9-item) DP (5-item) PA (8-item)	22	A seven-point Likert scale ranging from “never” (0) to “every day” (6)	74	Internal consistency: Overall (*α* = 0.81–0.94) EE (*α* = 0.62–0.96) DP (α = 0.15–0.98) PA (α = 0.60–0.92) Overall (*ω* = 0.78–0.88) EE (ω = 0.91) DP (ω = 0.64) PA (ω = 0.79) Test–retest reliability: *p* < 0.001 Intra Class Coefficient 0.77 Structural validity: EFA: factor loadings 0.55–0.86 CFA − 3 factors Convergent validity: Greater Spearman *r* between each item with its own dimension, and > 0.40 for all items Discriminant validity: Fornell-Larcker satisfied HTMT < 0.85–0.90	Internal consistency: EE (*α* = 0.86) DP (α = 0.74) PA (α = 0.57) Structural validity: 3 factors External validation (correlations between self-report and co-worker ratings): Emotional exhaustion (*r* = 0.41, *p* < 0.01) Depersonalisation (*r* = 0.57, *p* < 0.001) Discriminant validity (correlations between MBI and JDS – measure of job satisfaction): Emotional exhaustion (*r* = −0.23, *p* < 0.05) Depersonalisation (*r* = −0.22, *p* < 0.02) Personal accomplishment (*r* = 0.17, *p* < 0.05)
MBI-EE (9-item)	N/A	9	As above	11	Internal consistency: (α = 0.77–0.95) Structural validity: EFA: factor loadings 0.66–0.88	See MBI-HSS
MBI-EE (5-item)	N/A	5	As above	Not reported	Not reported	Not reported
MBI-EE (2-item)	N/A	2	As above	2	Internal consistency: (α = 0.67–0.83)	Not reported
MBI (2-item)	EE (1-item) DP (1-item)	2	As above	Not reported	Not reported	Concurrent validity: Single-item measure significantly related to overall MBI (*p* < 0.001)
aMBI	EE (3-tem) DP (3-item) PA (3-item)	9	As above	1	Internal consistency: EE (α = 0.73) DP (α = 0.69) PA (α = 0.61)	Not reported
MBI-GS	Exhaustion (5-item) Cynicism (5-item) Professional efficacy (6-item)	16	As above	29	Internal consistency: Exhaustion (α = 0.71–0.99) Cynicism (α = 0.71–0.97) Professional efficacy (α = 0.71–0.96) Structural validity: EFA – factor loadings > 0.72 CFA – 3 factors	Stability coefficients: Exhaustion (*r* = 0.65) Cynicism (*r* = 0.60) Professional efficacy (*r* = 0.67) Structural validity: 3 factors Factor loadings (lambda Y loadings *λ*): Exhaustion (0.69–0.82) Cynicism (0.53–0.89) Professional efficacy (0.51–0.70)
OLBI	Exhaustion (8-item) Disengagement (8-item)	16	A four-point Likert scale ranging from “totally disagree” (1) to “totally agree” (4)	20	Internal consistency: Overall (α = 0.76–0.92) Exhaustion (α = 0.73–0.90) Disengagement (α = 0.51–0.91) Exhaustion (ω = 0.79) Structural validity: CFA—mixed evidence with some supporting improved model fit for 2 factors and others supporting 4 factor model	Discriminant validity: Exhaustion and Disengagement (*r* = 0.44) Convergent validity: Exhaustion and Mental fatigue (*r* = 0.52, p < 0.05) Exhaustion and Satiation (*r* < 0.01) Disengagement and Satiation (*r* = 0.53, *p* < 0.05) Disengagement and Mental fatigue (*r* = −0.10, not statistically significant) Factor structure: 2 factors
CBI	Personal burnout (6-item) Work-related burnout (7-item) Client related burnout (6-item)	19	A five-point Likert scale ranging from “never”/ “very low” (0) to “always”/ “very high” (100) responses converted to 0–100 scale	46	Internal consistency: Overall (α = 0.86–0.94) Personal (α = 0.84–0.94) Work-related α = 0.79–0.93) Client-related (α = 0.77–0.91) Test–retest reliability: (*r* ≥ 0.70) Intra Class Coefficient = 0.954 Face validity index = 0.885 Content validity: S-CVI = 0.87 I-CVI = 0.67–1.00 CVR = 0.82 Structural validity: EFA – factor loadings > 0.541 CFA – 3 factors 3 factor models supported Rasch/ Item Response Theory: Person separation reliability = 0.85–0.90 Item reliability = 0.96–0.97 Good item fit ≥ 0.70 3 factor structure Limited DIF Convergent validity: AVE = 0.57; CR = 0.90 Discriminant validity (*n* = 1): Fornell–Larcker satisfied HTMT < 0.85 Measurement invariance: Configural, metric, and scalar invariance supported across exposure groups	Internal consistency: Personal (α = 0.87) Work-related (α = 0.87) Client-related (α = 0.85) Criterion validity: Personal burnout subscale and SF-36 vitality subscale showed the highest correlation Client-related burnout subscale and general health showed the lowest correlation
BMS	Measures dimensions including Physical exhaustion, Emotional exhaustion, and Mental exhaustion components of burnout as a single unidimensional construct	10	A seven-point Likert scale ranging from “never” (1) to “always” (7)	6	Internal consistency: (α = 0.89–0.97) (ω = 0.89)	Internal consistency: (α = 0.75) Structural validity: 1 factor
SMBM	Physical fatigue (6-item) Emotional exhaustion (3-item) Cognitive weariness (5-item)	11	A seven-point Likert scale ranging from “never”/ “almost never” (1) to “always”/ “almost always” (7)	5	Internal consistency: Overall (α = 0.94–0.96) Structural validity: EFA: all items loaded strongly on a single dominant factor, Eigenvalues > 3, supporting unidimensionality of construct	Internal consistency: Overall (α = 0.92) Physical fatigue (α = 0.92) Cognitive weariness (α = 0.87) Structural validity: 2 factors
CESQT	Job enthusiasm (5-item) Psychological exhaustion (4-item) Indolence (6-item) Guilt (5-item)	20	A five-point Likert scale ranging from “never” (0) to “very frequently: every day” (4)	1	Internal consistency: Job enthusiasm (α = 0.86) Psychological exhaustion (α = 0.81) Indolence (α = 0.80)	Internal consistency: Job enthusiasm (α = 0.76) Psychological exhaustion (α = 0.82) Indolence (α = 0.73) Guilt (α = 0.79) Structural validity: 4 factors
BBI	Work exhaustion (3-item) Cynicism (3-item) Sense of inadequacy (3-item)	9	A six-point Likert scale ranging from “completely disagree” (1) to “strongly agree” (6)	1	Internal consistency: Overall (α = 0.86) Work exhaustion (α = 0.65) Cynicism (α = 0.79) Sense of inadequacy (α = 0.72)	Internal consistency: Overall (α = 0.85) Work exhaustion (α = 0.70) Cynicism (α = 0.82) Sense of inadequacy (α = 0.71) Structural validity: 3 factors
Japanese Burnout Scale	Emotional exhaustion (5-item) Depersonalisation (6-item) Personal fulfilment (6-item)	17	A five-point Likert scale ranging from “never” (1) to “always” (5)	1	Internal consistency: Overall (α = 0.87) Structural validity: EFA supported 1 factor solution	Structural validity: 3 factors
Granada Burnout Questionnaire	Emotional exhaustion (9-item) Depersonalisation (7-item) Personal accomplishment (10-item)	26	A five-point Likert scale ranging from “total disagreement” (1) to “total agreement” (5)	Not reported	Not reported	Internal consistency: Emotional exhaustion (α = 0.93) Depersonalisation (α = 0.90) Personal accomplishment (α = 0.92) Structural validity: 3 factors
BAT-C	Exhaustion (8-item) Mental distance (5-item) Cognitive impairment (5-item) Emotional impairment (5-item)	23	A five-point Likert scale ranging from “never” (1) to “always” (5)	5	Internal consistency: Overall (α = 0.86–0.94) Exhaustion (α = 0.93) Mental distance (α = 0.85) Cognitive impairment (α = 0.86) Emotional impairment (α = 0.86) Structural validity: CFA – 4 factors	Internal consistency: Overall (α = 0.95) Exhaustion (α = 0.92) Mental distance (α = 0.91) Cognitive impairment (α = 0.92) Emotional impairment (α = 0.90) Factorial validity: The correlations between the factors ranged between 0.50 and 0.64 Discriminant validity: Good discriminant validity between BAT-C, MBI-GS and OLBI
COVID-19 Burnout Scale (10-item)	Measures dimensions including Physical exhaustion, Emotional exhaustion, and Mental exhaustion components of burnout as a single unidimensional construct	10	A five-point Likert scale ranging from “never” (1) to “always” (5)	2	Internal consistency: Overall (α = 0.94–0.97) Structural validity: EFA: factor loadings 0.62–0.89 CFA – unidimensional model Convergent validity: AVE = 0.88; CR = 0.97 Discriminant validity: Fornell-Larker satisfied HTMT < 0.85	Internal consistency: Overall (α = 0.92) Structural validity: 1 factor
COVID-19 Burnout Scale (13-item)	Emotional exhaustion (5-item) Physical exhaustion (4-item) Exhaustion due to measures against COVID-19 (4-item)	13	A five-point Likert scale ranging from “strongly disagree” (1) to “strongly agree” (5)	2	Internal consistency: Overall (α = 0.91) Emotional exhaustion (α = 0.88) Physical exhaustion (α = 0.87) Exhaustion due to measures against COVID-19 (α = 0.88)	Internal consistency: Overall (α = 0.92) Emotional exhaustion (α = 0.90) Physical exhaustion (α = 0.86) Exhaustion due to measures against COVID-19 (α = 0.86) Structural validity: 3 factors Factor rating range: 0.54–0.75
Multi-dimensional scales including a secondary subscale of burnout						
CFST	Compassion fatigue (23-item) Burnout (17-item)	40	A seven-point Likert scale ranging from “never” (0) to “always” (6)	Not reported	Not reported	Internal consistency: Overall (α = 0.86–0.94) Structural analysis: 1 stable burnout factor Structural reliability (stability) of this factor, as indicated by Tucker’s coefficient of congruence = 0.91
COPSOQ-II (full version contains 41 subscales across 7 major domains)	Demands at work (5 subscales) Work organisation and job content (8 subscales) Interpersonal relations and leadership (6 subscales) Work-individual interface (3 subscales) Values at the workplace (3 subscales) Health and wellbeing (8 subscales, which includes the 6-item Burnout subscale) Offensive behaviours (8 subscales)	127	A five-point Likert scale ranging from “never”/ “very low” (0) to “always”/ “very high” (100) responses converted to 0–100 scale	Not reported	Not reported	Internal consistency: Burnout subscale (α = 0.83)
Compassion Fatigue Short Scale	Burnout (6-item) Secondary traumatic stress (7-item)	13	A five-point Likert scale ranging from “rarely/never” (1) to “very often” (5)	1	Internal consistency: Burnout (α = 0.86)	Internal consistency: (α = 0.90)
ProQOL-5	Compassion satisfaction (10-item) Burnout (10-item) Secondary traumatic stress (10-item)	30	A five-point Likert scale Ranging from “never” (1) to “very often” (5)	31	Internal consistency: Burnout (α = 0.55–0.94) (ω = 0.83)	Internal consistency: Burnout subscale: α = 0.75 Discriminant validity: Shared variance between the subscales ranges from 2 to 34%
WBI	Measures domains including Burnout/ Emotional exhaustion, Fatigue, Depression, Distress/ Suicidal ideation, Stress/ Work-life balance, Quality of life, Meaning in work, Overall well-being	9	7 items – Dichotomous rating of “Yes”/ “No” 2 items (on Quality of life, and Meaning in work) – A five-point Likert scale Ranging from “never/ poor” (1) to “every day/ excellent” (5)	1	Internal consistency: Overall (α = 0.92)	Content validity index: 0.94 (relevance) 0.91 (representativeness) Sensitivity: Values ranged from 74–84% for Emotional exhaustion, and Depersonalisation Specificity: Values ranged from 72–78% for Emotional exhaustion, and Depersonalisation
PWLS	N/A	1	A five-point Likert scale ranging from “I enjoy my work. I have no symptoms of burnout” (1) to “I feel completely burned out and often wonder if I can go on. I may need help” (5)	1	Not reported	Convergent validity: Strong correlation with MBI-EE (*r* = 0.79)
Mini-Z Survey	Measures domains including Burnout (1-item) Key workplace drivers of burnout (9-item)	10	A five-point Likert scale scale labels differ per item	Not reported	Not reported	Internal consistency: Overall (α = 0.80) Construct validity: Drivers of the Mini-Z correlated at 0.26–0.46
Mini-Z (single-item)	N/A	1	A five-point Likert scale ranging from “no symptoms of burnout” (1) to “I feel completely burned out and may need help” (5)	Not reported	Not reported	Correlation with MBI-EE (*r* = 0.64)
PFI	Professional fulfilment (6-item) Work exhaustion (4-item) Interpersonal disengagement (6-item)	16	A five-point Likert scale ranging from “not at all true” (0) to “completely true” (4)	8	Internal Consistency: Overall (α = 0.84–0.92) Professional fulfilment (α = 0.74) Work exhaustion (α = 0.75–0.90) Interpersonal disengagement (α = 0.84–0.90)	Internal consistency: Overall (α = 0.92) Professional fulfilment (α = 0.91) Work exhaustion (α = 0.86) Interpersonal disengagement (α = 0.92) Convergent validity: Correlation between equivalent scales (PFI and MBI) range from 0.46–0.72 Test re-test reliability: Overall (*r* = 0.80) Professional fulfilment (*r* = 0.82) Work exhaustion (*r* = 0.80) Interpersonal disengagement (*r* = 0.71)

MBI-HSS, Maslach Burnout Inventory-Human Services Survey; MBI-EE, Maslach Burnout Inventory-Emotional Exhaustion; MBI-DP, Maslach Burnout Inventory-Depersonalisation; MBI-PA, Maslach Burnout Inventory-Professional Accomplishment; aMBI, Abbreviated Maslach Burnout Inventory; MBI-GS, Maslach Burnout Inventory-General Survey; OLBI, Oldenburg Burnout Inventory; CBI, Copenhagen Burnout Inventory; BMS, Burnout Measure-Short Version; SMBM, Shirom-Melamed Burnout Measure; CESQT, Spanish Burnout Inventory; BBI, Bergen Burnout Inventory; BAT-C, Burnout Assessment Tool-Core symptoms; CFST, Compassion Fatigue Self-Test; COPSOQ-II, Copenhagen Psychological Questionnaire-Version II; ProQOL-5, Professional Quality of Life-Version 5; WBI, Well-Being Index; PWLS, Physician Work Life Study; PFI, Professional Fulfilment index. N/A, Not Available.

Internal consistency, which is a psychometric property for reliability, was the most commonly reported psychometric property among all of the studies undertaken during the COVID-19 pandemic, being reported in all 19 burnout scales which had reported psychometrics (22, 23, 31–44). For Cronbach’s Alpha, coefficient values greater than 0.70 are indicative of adequate internal consistency (45). In this review, 15 burnout scales demonstrated adequate internal consistency within the COVID-19 context, as indicated either by their overall coefficients when available or by each relevant subscale coefficient reaching the accepted threshold. For four of the scales (MBI-HSS, Oldenburg Burnout Inventory (OLBI), BMS, ProQOL-5), reliability was also examined by the McDonald Omega coefficient, where 0.70 or higher are deemed as acceptable (46). In all four cases the values were above 0.70. For psychometric measures of stability over time, test–retest reliability and intraclass coefficients, were reported for MBI-HSS and Copenhagen Burnout Inventory (CBI). Both were found to be good stability over time, though the *r* coefficient was not reported for MBI-HSS.

Regarding psychometric properties for validity, 9 scales [MBI-HSS, MBI-EE (9-item), MBI-GS, OLBI, CBI, Shirom-Melamed Burnout Measure (SMBM), Japanese Burnout Scale, Burnout Assessment Tool-Core symptoms (BAT-C), COVID-19 Burnout Scale (10-item)], had reported validity information for the COVID-19 pandemic context. Structural validity was the most reported validity psychometric, being examined in 9 of the scales via confirmatory factor analysis to confirm the number of subfactors in seven scales. Rasch analysis which looks at whether a scale’s items function consistently as a construct over a range and across groups, was conducted only for one scale—CBI (47, 48). The CBI was also the only scale for which measurement invariance was reported, a psychometric which provides evidence for cross-cultural validity (49). Convergent and discriminant validities were reported together for three scales: MBI-HSS, CBI, and the COVID-19 Burnout Scale (10-item). Face and content validities were reported for the CBI, making it the burnout scale with the most psychometric properties documented.

In relation to the original validation article, psychometric properties were accessible and reported for 24 of the scales (22, 23, 30, 31, 33–44, 50–53). No prior psychometric properties were available for the three shortened versions of the MBI-HSS scales (MBI-EE: 2-item, MBI-EE: 5-item, aMBI). In the case of the MBI-EE (9-item), this was not-applicable as this scale represented the exact subscale from the full MBI-HSS version.

For 16 of these scales (22, 23, 31, 33–43, 50), internal consistency was reported through Cronbach’s alpha coefficients and all of these were reported as above 0.70, indicating good internal consistency. Stability and test–retest coefficients were reported for one scale each (41, 42). One scale reported (44) structural reliability as 0.91 according to Tucker’s Coefficient of Congruence (54).

For validity, at least one form of psychometric property was reported for 22 of the scales in the original validation articles. Structural validity was the most frequently reported, being reported in 12 of the scales, all indicating good fitting models and the expected number of subscales. Discriminant validity was reported for four of the scales (37, 39, 43, 50). Convergent validity was reported for four of the scales (30, 41, 50). Other validity properties reported included one scale for criterion and concurrent validity, respectively.

### 3.7 Most frequently used scales for burnout during the Covid-19 pandemic

We identified five scales that were frequently used in 40 or more studies. The most commonly used scale was Maslach Burnout Inventory-Human Services Survey (MBI-HSS) (37); which was used in 333 studies investigating burnout among healthcare workers during the COVID-19 pandemic. This comprises of three subscales: ‘emotional exhaustion’ (9-item), ‘depersonalisation’ (5-item) and ‘personal accomplishment’ (8-item).

The second most used scale was the Copenhagen Burnout Inventory (CBI) (34); which was used in 81 studies examining burnout among healthcare workers during the COVID-19 pandemic. This scale comprises of three subscales: ‘personal burnout’ (6-item), ‘work-related burnout’ (7-item), and ‘client-related burnout’ (6-item).

The third most used scale was Professional Quality of Life (ProQOL-5) (43) which is a multi-dimensional scale comprising of a secondary burnout subscale, and was used in 76 studies. This comprised of three subscales: ‘compassion satisfaction’ (10-items), ‘burnout’ (10-item), and ‘secondary traumatic stress’ (10-item). The two subscales of ‘burnout’ and ‘secondary traumatic stress’ make up the ‘compassion fatigue’ for this scale.

The fourth most used scale was the Maslach Burnout Inventory- General Survey (MBI-GS) (42); which was used in 60 studies. This scale comprises three subscales: ‘exhaustion’ (5-item), ‘cynicism’ (5-item), and ‘professional efficacy’ (6-item).

The fifth most used burnout scale was the Oldenburg Burnout Inventory (OLBI) (32); which was used in 40 studies. This scale comprises two subscales: exhaustion (8-item) and disengagement (8-item).

## 4 Discussion

### 4.1 Key findings from the review

Burnout among healthcare workers has been extensively assessed during the COVID-19 pandemic, as evident from the substantial number of research articles included in our review. A total of 27 scales were used to measure burnout across various roles among healthcare workers during the pandemic. The majority of scales (*N* = 25) were developed and validated prior to the COVID-19 pandemic. Only two scales were developed and their psychometrics examined for the first time during the COVID-19 pandemic (24, 55), however one of these scales represented a previously used burnout scale which had been adapted for the COVID-19 context without further significant modifications (23). More than half of the scales (*N* = 15) were originally developed in the United States.

The majority of the articles included in this systematic review reported on the reliability of the scales but only a third of the scales had any reported validity measures during the COVID-19 pandemic. Consequently, for the majority of the burnout scales, we cannot be certain whether the factor structure, construct validity, or convergent/ discriminant validities would still apply in the COVID-19 context. Both reliability and validity psychometrics were available for MBI-HSS, MBI-EE (9-item), MBI-GS, OLBI, CBI, SMBM, Japanese Burnout Scale, BAT-C, and COVID-19 Burnout Scale (10-item) (23, 34, 36, 37, 39, 40, 42, 50). None of the multi-dimensional scales which secondarily measured burnout had reported validity psychometrics.

When examining these scales and their psychometrics, 15 scales exhibited desirable properties in terms of having good internal consistencies. For the most commonly used scale – MBI-HSS, out of the 74 studies that used MBI-HSS and provided psychometric properties during the COVID-19 pandemic, 11 studies reported alpha values ranging from 0.58 to 0.68. While these values are lower than the conventional 0.70 threshold, some researchers have suggested that lower values can still be considered satisfactory, with the alpha value of 0.58 being considered satisfactory by Gambaro et al. (56). However, it must be noted that three studies reported alpha values of 0.15–0.56 with the markedly low value of 0.15 being reported by one study for the ‘depersonalisation’ subscale (57).

For eight of the scales measuring burnout identified in this review, there were no studies conducted during the COVID-19 pandemic which had reported on their psychometric properties. These scales were as follows: MBI-EE (5-item), MBI-EE/DP (2-item), Mini-Z Survey, Mini-Z (1-item), PWLS, COPSOQ-II, CFST, and Granada Burnout Questionnaire. Out of these 8 scales, psychometric properties from the original validation articles were available for all scales except for the MBI-EE: 5-item scale.

For 24 of the burnout scales, psychometric properties were available in the original validation articles. Three scales had no prior psychometrics available which were: MBI-EE (5-item), MBI-EE (2-item), and aMBI. Hence, MBI-EE (5-item) was the only scale which had no psychometric data available at all prior or during the COVID-19 pandemic.

From the results of this review, 19 of the 27 burnout scales had at least one reported psychometric property during the COVID-19 pandemic. By and large, this focused on reliability measures with internal consistency being reported for all scales and test–retest reliability for two scales. Only a third of the burnout scales had reported measures for validity, and this was largely limited to structural validity. This finding is significant as validity metrics, including measurement invariance to establish cross-cultural validity, are crucial for determining whether a scale performs consistently across different groups and settings, which was only present for the CBI scale. This is particularly important in the COVID-19 context, given that the pandemic affected countries differently as a result of diverse political responses and public health strategies. The details of the country in which each scale was developed may also help explain differences in attention given to burnout among healthcare workers during the COVID-19 pandemic across countries. The predominance of scales originally developed in the United States of America may also contribute to discrepancies when applied in other national contexts.

### 4.2 Comparisons with other literature

A scoping literature review conducted on the psychometric properties of burnout instruments on healthcare workers during the COVID-19 pandemic (58) identified only seven burnout instruments (also included in our review) (34, 36, 37, 39, 40, 42, 50). Moreover, their search was limited to burnout instruments that did not include any other subscales measuring different constructs, with their search being conducted in the early stages of the pandemic from January 2020 until November 2021. Consequently, our research was able to reveal a much broader array of burnout instruments, as our search encompassed articles published from the onset of the pandemic through to March 2023. Furthermore, this systematic review identified a substantially larger number of eligible articles (*N =* 794) compared to the former scoping review (*N* = 31).

A systematic review on the psychometric properties of burnout measures published before the COVID-19 pandemic examined the validity of five burnout scales used to assess occupational burnout in mental health workers (20). Three of these scales were also included in our review (34, 37, 50). For two of these scales (CBI, MBI-HSS), the Cronbach’s alphas were comparable, indicating that the internal consistencies were satisfactory in both COVID-19 and non-COVID-19 contexts (the previous review did not report upon the reliability of the OLBI). Regarding validity comparisons, in this earlier review, the factor structure of the CBI scale was not reported. However, we identified that the three-factor structure of this scale held in the COVID-19 context. Moreover, there were mixed findings regarding the factor structure of the MBI-HSS scale in the previous review, whereas in our review we identified support for the factor structure of the MBI-HSS in the COVID-19 context. For OLBI, the factor structure results were also comparable between the previous review and this review, indicating a good fit for the two-factor model, although the data for this came from the original validation article in this review.

### 4.3 Strengths and limitations

One of the key strengths of this systematic review is our extensive search process, which involved gathering a large pool of articles from various databases and not limiting our search to any language restrictions. This enabled us to screen and analyse peer-reviewed articles from around the world, providing a deeper understanding of the burnout scales used during the COVID-19 pandemic in different countries, along with their psychometric properties in this novel context. Furthermore, our search was not restricted to specific burnout scales, allowing us to gather more information on the frequently used scales, the countries where they were used, and even identify new burnout scales developed during the COVID-19 pandemic. Additionally, our search criteria encompassed all healthcare job types listed by the World Health Organisation (WHO, 2019), enabling us to capture the burnout research conducted across the entire healthcare profession during the pandemic.

The main limitation of this systematic review is that we did not perform a risk of bias assessment for each study due to extracting a large quantity of articles (794 records), and we did not undertake a standardised appraisal of the included measures for quality assessment. However, our search strategy was particularly over-inclusive, and we were able to systematically review and report the psychometric properties of included scales both during COVID-19 and before COVID-19, providing a comprehensive overview of their use and quality. Furthermore, while we compared the psychometric properties of the COVID-19 studies with those from the original validation articles, we did not examine the multitude of studies that have been published in the interim focusing on burnout scales. Consequently, we were unable to compare these psychometric properties with other validation studies that were published between the year of scale development and the time of the COVID-19 pandemic.

### 4.4 Implications for future practice and research

This review identified the scales used to assess burnout during the COVID-19 pandemic and their psychometric properties, through which it can inform researchers and healthcare workers when selecting a scale to measure burnout.

A gold standard scale to assess burnout during the pandemic has not been established, and most of the burnout scales used were developed before the pandemic. The psychometric properties of some of these scales (*N* = 19) were assessed during the COVID-19 pandemic, for which prior psychometrics were available in their validation articles for most of them (*N* = 16). However, the type of psychometric properties assessed varied from scale to scale, and the internal consistency was the predominant psychometric property reported (*N* = 19). Thus, among the identified scales, it is advisable to select one that has been evaluated for both reliability and validity during the pandemic. Given the relative limited availability of validity metrics identified in this review, future research on burnout scales should prioritise rigorous validity assessment of these instruments.

In application of the above principles for the COVID-19 context, CBI had the most psychometric properties available during this period, while MBI-HSS was the most popular scale used to measure burnout. Other notable scales with desirable psychometric properties include the SMBM, and the two COVID-19 Burnout Scales which all had high internal consistency ranges above 0.90. However, this was limited by the relative low number of studies reporting on the psychometrics as well as their use determined by different factors for burnout dimensions being investigated. For instance, SMBM and BAT-C uniquely measure ‘cognitive impairment/weariness’ dimension of burnout whereas the COVID-19 Burnout Scale (Galanis et al.) measures the novel ‘exhaustion due to measures against COVID-19’ dimension.

Furthermore, the choice of the scale would also depend on several other factors, most notably the goal of the assessment, the type of healthcare workers on whom burnout is to be assessed, the language of the assessment, and the time available for it.

If the goal is to assess burnout in international studies, comparing scores with other data, or comparing burnout levels in different health workers, then the MBI-HSS would be the first choice of recommendation. While scales, such as the SMBM, present more robust psychometric properties, especially regarding internal consistency, the CBI has been tested for more psychometric properties that are also satisfactory, both during and before the pandemic, whereas the MBI-HSS is more likely to be familiar and well-known. It also generally represents the most widely used measure of burnout, available in multiple languages and applied to various healthcare workers. If one were to be interested in a specific aspect of burnout (like emotional exhaustion), then shorter versions of MBI-HSS are also available for this. However, data on their psychometric properties are scarce, and their use against the full version of the scale should be balanced well.

If the pragmatic part of the assessment is a priority, and using a shorter scale is preferred, then using a scale like the BMS can be a satisfactory option. It consists of 10 items, is validated in different languages, can be used by different healthcare workers and shows good psychometric properties.

Moreover, if burnout is being considered as one component of a composite index, then multi-dimensional scales such as the ProQOL-5 might represent a good option. However, burnout measured within such composite scales place different emphasis on certain dimensions of burnout, hence caution would be advised to look into the burnout items being assessed.

There is no evidence suggesting the use of different scales for different healthcare workers. If burnout is to be assessed in a specific health profession, it is recommended to use any one of the above-mentioned scales.

The findings of this systematic review could be useful for those involved in developing and implementing regular surveys for healthcare workers, such as the National NHS Bank Survey (United Nations). Current surveys already enquire about wellbeing-related issues, and the resulting data inform policy decisions. Incorporating a validated and reliable burnout scale from those identified in this review could enhance the comprehensiveness of future surveys for this purpose. Given the large number of instruments identified for measuring burnout in this review, there does not appear to be a need to develop additional scales, as existing options seem well-suited for assessing burnout, including in the novel context of the COVID-19 pandemic. Since the pandemic placed unprecedented pressure on healthcare workers, it is plausible that the findings from this review also apply to other emergency crises that impose significant strains on healthcare workers.

## 5 Conclusion

There has been extensive research interest in burnout among healthcare workers during the COVID-19 pandemic. This systematic review identified a large number of quantitative studies that assessed burnout in healthcare workers using a variety of self-report instruments. All but two of these scales had been validated prior to the COVID-19 pandemic. Psychometric properties were reported for 19 scales, however this was predominantly on reliability rather than validity. Consequently, this review has suggested avenues for future research and practice, emphasising priority for rigorous validity assessment in future burnout research, and outlining how the findings from this review can be used to further inform policy and practice in novel contexts such as the COVID-19 pandemic.

## Data Availability

The original contributions presented in the study are included in the article/supplementary material, further inquiries can be directed to the corresponding author.
